# Immune response in honey bees (*Apis mellifera)* following humic substance feeding

**DOI:** 10.1007/s11259-026-11360-8

**Published:** 2026-06-18

**Authors:** Lenka Kollár Moskáľová, Natália Chomová, Marek Ratvaj, Rastislav Sabo, Jiří Danihlík, Silvie Dostálková, Bisa Saraswathy, Mette Sørensen, Md Abdul Latif, Youngjin Park, Dagmar Mudroňová

**Affiliations:** 1 Department of Microbiology and Immunology, University of Veterinary Medicine and Pharmacy, Košice, 04181 Slovakia; 2 Department of Pharmacology and Toxicology, University of Veterinary Medicine and Pharmacy, Košice, 04181 Slovakia; 3 Department of Biochemistry, Faculty of Science, Palacký University Olomouc, Olomouc, 77900 Czechia; 4 Faculty of Biosciences and Aquaculture, Nord University, Bodø, 8026 Norway; 5 Department of Aquatic Life Medical Sciences, Sunmoon University, Asan, 31460 Republic of Korea

**Keywords:** Bee immunity, Humic substances, Antimicrobial peptides, Antioxidant status, Hemocytes

## Abstract

Humic substances (HS) are increasingly investigated as natural additives to support health in humans and animals, yet their effects on honey bees remain insufficiently characterised. This study evaluated the impact of a commercial HS-based product on immunity and oxidative status of *Apis mellifera*. Newly emerged worker bees were kept under laboratory conditions and fed sugar syrup supplemented with 0.5% HS for seven days, while the control group received unsupplemented syrup. Effects of HS were evaluated by analysing the expression of selected immune-related genes, quantifying antimicrobial peptides (AMPs), measuring the activity of antioxidant enzymes, evaluating lipid peroxidation, and characterising hemocyte populations. HS supplementation significantly increased relative gene expression of *superoxide dismutase 1* and *prophenoloxidase*, while expression of genes for *apidaecin* and *defensin 1* decreased. HS supplementation significantly increased relative gene expression of *superoxide dismutase 1* and *prophenoloxidase*, while expression of genes for *apidaecin* and *defensin 1* decreased. Similarly, catalase enzymatic activity was significantly decreased in the HS group. No significant changes were detected in gene expression for other AMPs and relative abundance of AMPs, or other antioxidant parameters. HS supplementation significantly increased the proportion of medium granular hemocytes (*p* < 0.05) at the expense of high granular cells (*p* < 0.01). In conclusion, the 7-day administration of HS at 0.5% did not adversely affect bee health and modulated selected immune parameters. The biological significance of these immunomodulatory effects requires confirmation through challenge studies and field trials under natural conditions.

## Introduction

In the EU, approximately 84% of crops depend on insect pollination, contributing between €5 and €15 billion annually, and without pollinators, food production would be seriously threatened (EEA 2025). Pollination is widely recognised as a fundamental ecosystem service and a cornerstone of agricultural productivity, with bee-pollinated crops contributing substantially to global food supply and crop quality (Khalifa et al. 2021). Therefore, the honey bee (*Apis mellifera*) makes an important contribution not only to ecosystem stability but also to plant species diversity (Papa et al. 2022). Moreover, honey bees and their products find widespread application worldwide across various fields, such as food production, religious rituals, and human and veterinary medicine (Tafere 2021; Balasubramanyam 2024; Stevanović et al. 2024). Honey bees are affected by various biotic and abiotic stressors; however, they can mitigate the impact of these stress factors through activation of their immune system, which responds to environmental and biological challenges (Neov et al. 2019; Morfin et al. 2021).

Individual immunity in honey bees can be generally divided into the physical body barriers, cellular immunity mediated mainly by hemocytes, which includes processes such as phagocytosis, nodulation, encapsulation, or melanisation, and humoral immunity, involving the activities of phenoloxidase reaction, lysozyme, and antimicrobial peptides (AMPs). In addition to their individual defences, honey bees demonstrate social immunity. This includes behaviours like brood care and hygienic activities, also found in solitary and subsocial bees, and social fever, where the nest temperature is raised collectively to combat pathogen challenges (Morfin et al. 2021; Hurychová et al. 2024).

The European Union prohibits the use of antibiotics and sulfonamides for the treatment or prevention of infectious diseases in beekeeping (Rodrigues et al. 2024). Consequently, there is an increasing focus on the investigation and application of natural substances (e.g. probiotics, prebiotics, phytoadditives, humic substances) for both treatment and prevention, as well as for stimulating the overall health and immunity of honey bees (Kuzyšinová et al. 2016).

Humic substances (HS) are a structurally complex group of compounds naturally found in soil. They represent a heterogeneous organic material of high molecular weight formed through the transformation of residues from living organisms into organic compounds in a process known as humification (Yang et al. 2021; Vikram et al. 2022). HS primarily consist of humic acid, humus, ulmic acid, fulvic acid, humin, and various trace elements (Arif et al. 2019). HS are employed not only in human medicine but also in veterinary medicine. In veterinary applications, HS and their fractions exhibit a wide range of biological activities, including detoxification, anti-inflammatory, antiviral, antimicrobial, antioxidant, immunomodulatory, and hepatoprotective effects, and improved production performance (Islam et al. 2005; Kovacik et al. 2020; Mudroňová et al. 2021; He et al. 2023; Hriciková et al. 2023; Huang et al. 2023; Xu et al. 2023).

Recent research on HS in relation to honey bees has primarily focused on evaluating the effects of humic compounds, their specific fractions, and various formulations on the reproductive performance of bees (Rumyantsev et al. 2023a), on extending their lifespan and improving physiological condition (Rumyantsev et al. 2023b), as well as on brood development, colony productivity, and honey yield (Tunç et al. 2021). Furthermore, several studies have explored the potential application of HS in controlling *Varroa destructor* infestations (Yilmaz and Dizman 2023).

Although there is considerable knowledge about the effects of HS on immunity in various animal species, their effects on honey bees’ health remain under-researched. As a result, this study investigates the effect of a commercially available HS supplement in the diet of the honey bee. By analysing immune response, antioxidant enzymes, and hemocyte populations, we aimed to determine whether this dietary additive offers benefits for honey bee health.

## Materials and methods

### Experiment design

**Fig. 1:**
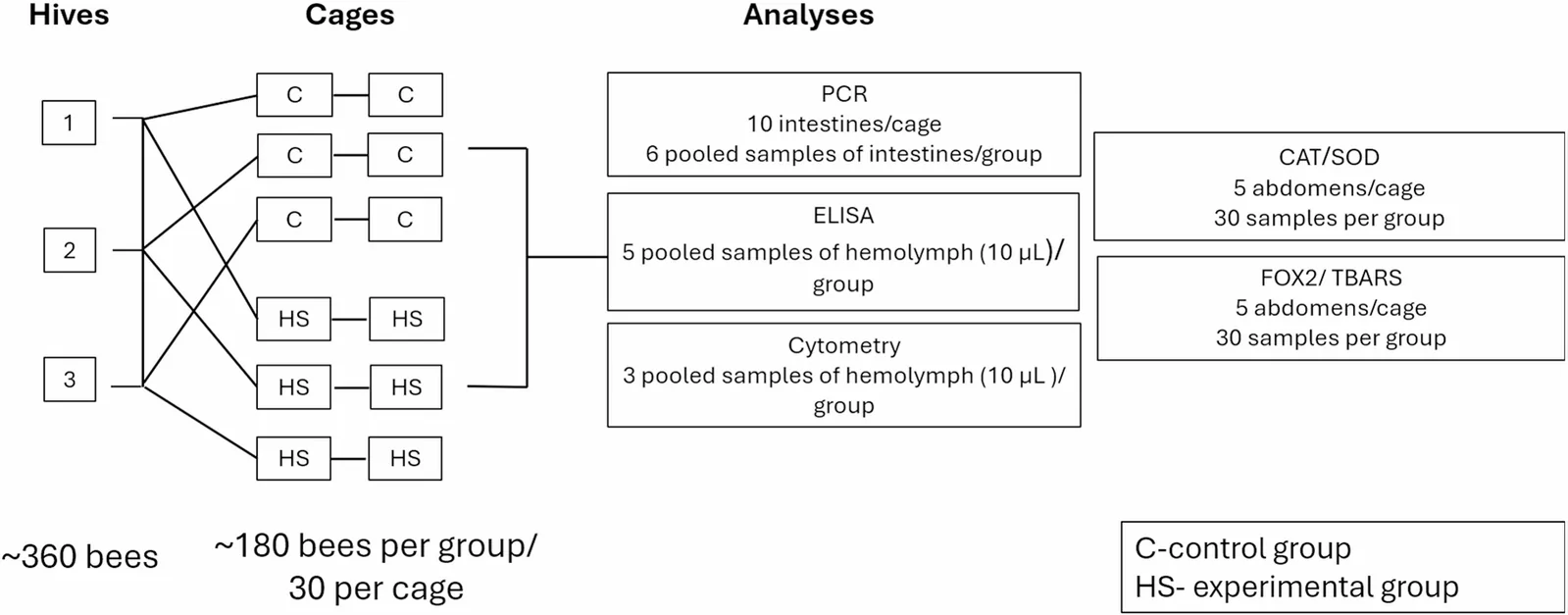
A scheme of division of bees into groups and the samples collected for analyses. The detailed process of sample collection is described in the respective sections

To minimise the age-dependent variability in the experiment, only newly emerged workers (within 24 h) were used. Capped honey bee (*Apis mellifera*) brood was collected from three different hives maintained at the University of Veterinary Medicine and Pharmacy in Košice, Slovakia, and hatched under laboratory conditions. The bees in the source colonies were in good health condition, without clinical signs of disease, as assessed by experienced beekeepers. After hatching, the bees (*n* = 360) were randomly divided into the control (CTRL) and the experimental group (HS group). Bees were divided into 6 steel cages per group with 30 individuals per cage (Fig. 1), to ensure sufficient space and capacity for feeding, and kept at 34 °C and 60–70% relative humidity. The experimental group was fed sugar syrup (50% (w/v) sucrose solution) supplemented with a commercial product containing HS from leonardite at a final concentration of 0.5% (v/v) (HUMAC^®^ Natur AFM Liquid, Humac s.r.o., Košice, Slovakia). The HS used were in liquid form and specifically designed by the manufacturer for bees. This concentration of HS was chosen based on the manufacturer’s recommended dosage. The control group received only sugar syrup. For feeding, perforated 1.5 mL microcentrifuge tubes were used to minimise evaporation. Fresh syrup was provided daily, and the bees were fed *ad libitum*.

After 7 days of supplementation, bees were anesthetised with CO₂. Hemolymph was collected by puncturing the thorax using a glass capillary, and bees were then euthanised by decapitation and dissected. Intestines and abdomens were separated and processed as described in the respective methodological sections below.

### Composition of humic substances as specified by the manufacturer

The composition as specified by the manufacturer includes natural humic acids in the liquid form at a minimum of 15% and in the dry matter at a minimum of 45%, with fulvic acids not exceeding 5%. The content of minerals is as follows: calcium (Ca) 1200 mg/L, magnesium (Mg) 55 mg/L, iron (Fe) 260 mg/L, copper (Cu) 1.70 mg/L, manganese (Mn) 1.97 mg/L, selenium (Se) 0.077 mg/L, vanadium (V) 4.85 mg/L, zinc (Zn) 2.65 mg/L, molybdenum (Mo) 0.295 mg/L, and sodium (Na) 10,050 mg/L. Additionally, all naturally occurring trace elements are present in µg/kg and bound within a carboxymethylcellulose complex of organic matter. The product contains all-natural amino acids that primarily facilitate the bioavailability of trace elements. Moisture content is a maximum of 70%, pH ranges from 8.0 to 8.3, and particle size varies between 0.01 and 50 μm.

### Relative gene expression analysis by qPCR

Relative gene expression was analysed from 10 homogenised bee intestines per cage, a total of 6 pooled samples per group. After dissection, the intestines were cleaned, washed in sterile phosphate-buffered saline (PBS), and placed in RNAlater stabilisation solution (Thermo Fisher, Lithuania), and stored at -20 °C until RNA extraction. Pooled samples were homogenised using 1.4 mm zirconia beads (Bertin Technologies, France) and 400 µL TRK lysis buffer (Omega Bio-tek, USA) in a Precellys 24 homogeniser (Bertin Technologies, France), using two 30 s cycles at 6,000 rpm with a 15 s pause between cycles. Total RNA was extracted from intestinal samples using the Omega E-Z Total RNA Kit (Omega Bio-tek, USA) according to the manufacturer’s protocol. The purity and concentration of RNA were measured with Nanodrop 8000 (Thermo Scientific, USA). Following RNA isolation, complementary DNA (cDNA) was synthesised from 1 µg RNA using the QuantiTect Reverse Transcription Kit (Qiagen, Germany), which also includes a gDNA removal step. Quantitative PCR (qPCR) was performed using Luna^®^ Universal qPCR Master Mix (New England Biolabs, USA) following the manufacturer’s instructions on a CFX96 Real-Time PCR Detection System (Bio-Rad, USA). The protocol consisted of initial denaturation at 95 °C for 60 s and 40 cycles of denaturation at 95 °C for 15 s, followed by extension at 60 °C for 30 s. Reactions consisted of 5 µL of Luna Master Mix, 4 µL of cDNA (10 ng/µL), and 0.3 µL of each primer (10 µM stock, final concentration 300 nM) with water up to a final volume of 10 µL. Each reaction was performed in triplicate. Each qPCR reaction included a no-template control and a series of standard dilutions of pooled cDNA. The amplification efficiency was 100% ± 10%. The specificity of products was confirmed by a melting curve analysis. The primers used in the reactions are listed in the corresponding table (Table 1). Relative gene expression was calculated using the ΔΔCt method (± standard deviation) with CFX96 Manager Software (Bio-Rad, USA). The reference genes used for normalisation were *gapdh*, *rps18*, and *rpl13a*. The stability of reference genes was confirmed using the Reference Gene Selection Tool in BioRad Software.

**Table 1:** List of primers used in RT-qPCR for immune-related genes

Primer	Forward	Reverse	Reference
*abaecin*	CGCACTACTCGCCACGATAT	TCGGATTGAATGGTCCCTGAC	(Li et al. 2022)
*apidaecin*	TTTTGCCTTAGCAATTCTTGTTG	GAAGGTCGAGTAGGCGGATCT	(Shi et al. 2018)
*defensin 1*	TGCGCTGCTAACTGTCTCAG	AATGGCACTTAACCGAAACG	(Tesovnik et al. 2020)
*hymenoptaecin*	CTCTTCTGTGCCGTTGCATA	GCGTCTCCTGTCATTCCATT	(Evans et al. 2006)
*superoxide dismutase 1 (sod 1)*	AGCAGATGCAAGTGGTGTTG	GAGCACCAGCATTTCCTGTAG	(Collins et al. 2004)
*superoxide dismutase 2 (sod 2)*	GTCGCCAAAGGTGATGTCAATAC	CGTCTGGTTTACCGCCATTTG	(Li et al. 2014)
*thioredoxin peroxidase 3 (tpx 3)*	CTGCACCTGAATTTTCCGG	CCTTTGTAATCACTTAATTTGATTTCTT	(Corona et al. 2005)
*relish*	GCAGTGTTGAAGGAGCTGAA	CCAATTCTGAAAAGCGTCCA	(Khongphinitbunjong et al. 2015)
*prophenoloxidase (proPO)*	ACAGATCCTGTATGGATTGC	TCTTGGACGAGTAAACGAT	(Zaobidna et al. 2015)
*glutathione S-transferase (gst)*	AGGAGAGGTGTGGAGAGATAGTG	CGCAAATGGTCGTGTGGATG	(Li et al. 2014)
*gapdh*	GATGCACCCATGTTTGTTTG	TTTGCAGAAGGTGCATCAAC	(Scharlaken et al. 2008)
*rps18*	GATTCCCGATTGGTTTTTGA	CCCAATAATGACGCAAACCT	(Scharlaken et al. 2008)
*rpl13a*	TGGCCATTTACTTGGTCGTT	GAGCACGGAAATGAAATGGT	(Scharlaken et al. 2008)

### Relative quantification of abaecin, hymenoptaecin, and defensin 1 by the ELISA

The antimicrobial peptides abaecin, defensin 1, and hymenoptaecin were quantified using an enzyme-linked immunosorbent assay (ELISA) as described by Hurychová et al. (2024) and Pinďáková et al. (2025). Five pooled hemolymph samples of 10 µL hemolymph were collected from bees for each treatment group and diluted 10× with 0.1% trifluoroacetic acid. Hemolymph was lyophilised and then dissolved in 600 µL of ELISA coating buffer. Briefly, a total of 100 µL of diluted hemolymph samples, blanks (coating buffer), positive controls (synthetic peptide epitopes), negative controls (hemolymph from newly emerged bees), and calibrators were added to each well and incubated overnight at 4 °C for antigen binding.

After antigen binding, primary antibodies for abaecin, defensin 1, and hymenoptaecin (sourced from Clonestar Peptide Services, Czech Republic) were added. The antibodies were diluted as follows: 1:250 for abaecin, 1:500 for defensin 1, and 1:500 for hymenoptaecin. Plates with primary antibodies were incubated for 1 h at 37 °C, followed by washing. The secondary antibody, goat anti-rabbit IgG (whole molecule)-peroxidase conjugate (Sigma-Aldrich, USA), was then added at a dilution of 1:3000 in washing buffer and incubated for 1 h at 37 °C. After the washing, the reaction was developed using tetramethylbenzidine substrate in 0.1 M phospho-citrate buffer with sodium perborate (4 mM); the plates were incubated in the dark for 1 h at 37 °C. The reaction was stopped by the addition of 0.5 M H_2_SO_4_. Absorbance was measured at 450 nm using a Synergy HT microplate reader (BioTek, USA). Epitopes for individual AMPs were used as standards for positive control. Each reaction was performed in duplicate.

### Quantification of lipid peroxidation by Ferrous oxidation in xylenol orange (FOX2) and Thiobarbituric acid reactive substances assay (TBARS) methods

The level of lipid peroxidation in the abdomen of bees, without the digestive tract, was measured using the FOX2 and TBARS methods, as described by Kunc et al. (2023) and Pinďáková et al. (2025). Dissected abdomens (5 per cage; 30 per group) were individually homogenised in liquid nitrogen using 3-mm glass beads (Roth, Germany), after which 750 µL of 80% ethanol containing 0.01% butylhydroxytoluene was added to each homogenate. After homogenization, the samples were centrifuged at 16,000 × g for 10 min at 5 °C. The supernatant was then separated, transferred to new vials, and stored on ice. This sample preparation method was identical for both the FOX2 and TBARS analyses.

Lipid hydroperoxide quantification in bees was performed using a protocol based on the FOX2 method described by Sodergren (1998) with modifications described by Kunc et al. (2023). Absorbance was measured at 560 nm on a Synergy H1 Multi-Mode Reader (BioTek Instr., USA), and the lipid hydroperoxide content was expressed as the cumene hydroperoxide equivalent concentration based on the calibration curve. Each reaction was performed in triplicate.

Malondialdehyde (MDA), a final product of lipid peroxidation, was quantified using the TBARS method Hodges et al. (1999) with modifications by Kunc et al. (2023). Fluorescence was measured at an excitation wavelength of 525 nm and emission at 560 nm using the Synergy H1 Multi-Mode Reader (BioTek Instr., USA). MDA content was determined based on the difference in fluorescence between the samples and the blanks, where the MDA-TBA2 product was formed. MDA was quantified based on a calibration curve, using 1,1,3,3-tetraethoxypropane as a standard. Each reaction was performed in triplicate.

### Determination of catalase activity, superoxide dismutase, and protein concentration

For analysis of antioxidant enzymes, only abdomens without the digestive tract were used. Dissected abdomens (5 abdomens per cage, 30 abdomens per group) were individually homogenised in liquid nitrogen using glass beads (3 mm in diameter, Roth, Germany). Then 200 µL of 0.1 M potassium phosphate buffer, pH 7.0, was added to the homogenate. Samples were centrifuged for 10 min at 12,000 × g and 4 °C. The supernatant was used for further measurements. For all samples, protein concentration was determined according to Bradford (1976) at 595 nm with modifications by Dostálková et al. (2022). Each reaction was performed in triplicate.

Determination of catalase activity (CAT) was performed as described by Dostálková et al. (2022). Extracts from bee abdomens were diluted 1:2 in potassium phosphate buffer at pH 7.0. The enzymatic reaction was measured according to the method of Aebi (1984) using a Synergy H1 Multi-Mode Reader at 240 nm (BioTek Instr., USA).

The reaction mixture consisted of 220 µL of phosphate buffer and 10 µL of the sample, with the reaction initiated by adding 20 µL of 150 mM H_2_O_2_ (Lach-Ner, Czech Republic). Changes in absorbance at 240 nm were recorded over a 5-minute period at 30 °C. Catalase activity was expressed as the amount of H_2_O_2_ decomposed, using a molar extinction coefficient of ε_240_ = 39,400 M^− 1^·cm^− 1^, per 1 mL of bee extract.

Determination of superoxide dismutase activity (SOD) was determined as described by Dostálková et al. (2022). Extracts from bee abdomens were diluted 1:50 in 0.02 M Tris buffer (pH 8.2) containing 1 mM EDTA. The activity was measured according to the method described by Marklund and Marklund (1974) at 320 nm.

The reaction mixture consisted of 220 µL of Tris buffer and 20 µL of the diluted sample. To initiate the reaction, 20 µL of 2 mM pyrogallol was added to the well. Absorbance changes at 320 nm were monitored for 5 min at 25 °C. Each reaction was performed in triplicate.

SOD activity was expressed in enzyme units (U) per millilitre of bee extract. One unit is defined as the amount of enzyme required to inhibit pyrogallol auto-oxidation by 50%, as described by Marklund and Marklund (1974).

### Statistical evaluation of results

Statistical evaluation was performed with JASP version 0.19.0 (JASP Team, University of Amsterdam, Netherlands). In the first step, the normality of the data was tested using the Shapiro-Wilk test. In cases where the data did not exhibit a normal distribution, they were transformed using a logarithmic transformation. Subsequent statistical analyses were performed on the transformed data. In case of normal data distribution, the difference between the control and experimental groups was compared using an unpaired Student’s t-test. In the case of non-parametric data, the Mann-Whitney test was used.

### Imaging flow cytometric analysis of hemocytes

Hemolymph was collected from anesthetised bees by puncturing the dorsal sinus of the thorax using a thin glass capillary. Three pooled hemolymph samples of 10 µL were collected from bees for each treatment group. Hemocytes for subsequent imaging flow cytometry analysis were labelled with the nuclear dye Hoechst 33,342 (Sigma, USA) as follows: 10 µL of hemolymph was diluted with 40 µL of PBS (MP Biomedicals, France) and stained with 4 µL of the Hoechst 33,342 (0.25 µg/µL) for 5 min at room temperature in the dark.

**Fig. 2:**
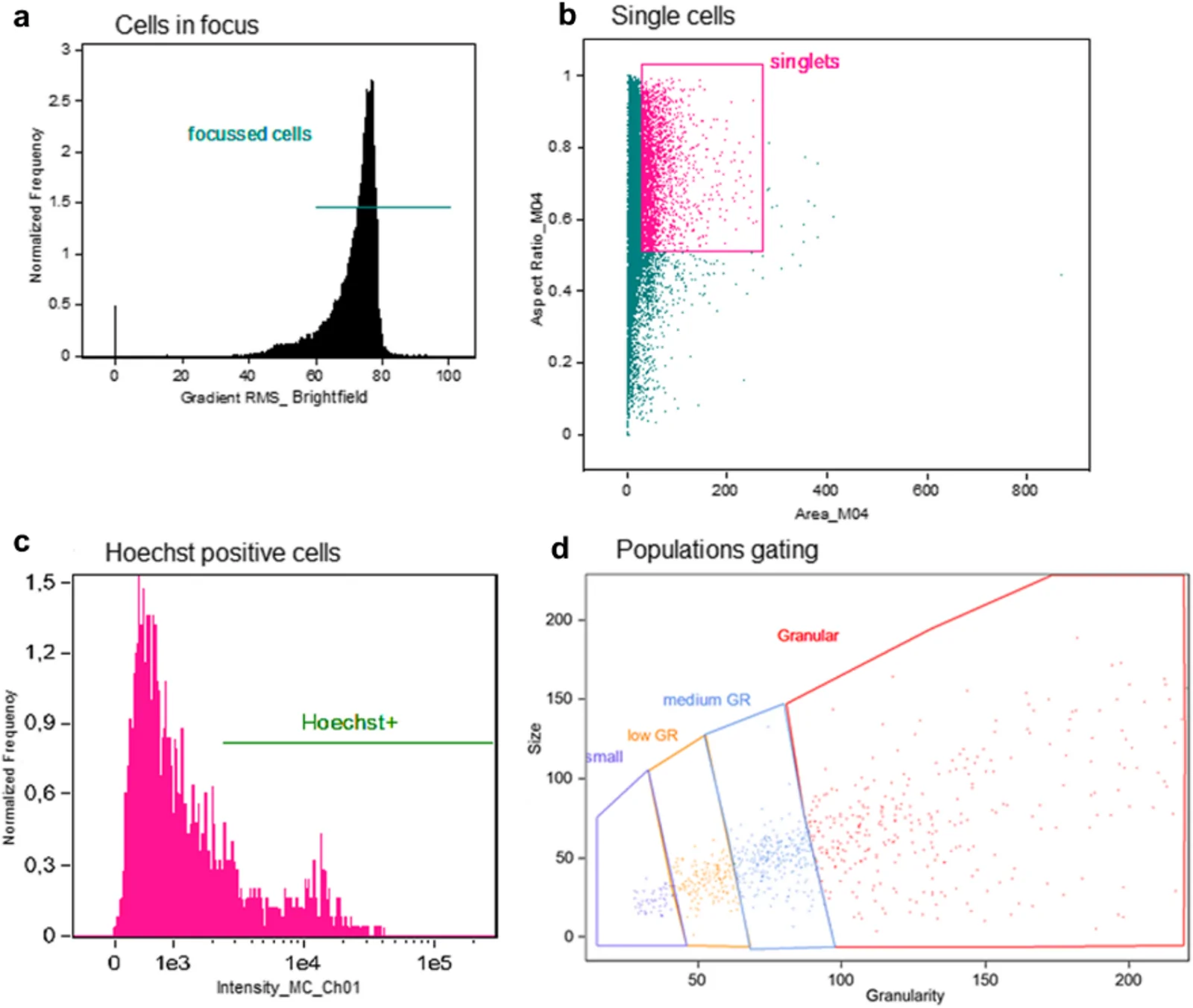
Gating strategy used for bee hemocyte analysis: (**a**) Cells in focus were gated by the help of IDEAS gradient RMS feature which selects high-quality images (the value above 60 shows high focused images); (**b**) gating of single cells (area indicates cell size while aspect ratio means a ratio of vertical and horizontal length of cell diameter); (**c**) selection of Hoechst positive cells; (**d**) division of Hoechst+ cells to: small and very low granular cells – violet, low granular cells – orange, medium granular cells – blue and high granular cells – red

The flow cytometric analyses were performed using an ImageStream^®^X Mk II Imaging Flow Cytometer (Luminex Corporation, USA) equipped with two argon-ion lasers (488 nm and 642 nm), and a side scatter laser (785 nm). Cell population and morphology analysis using imaging flow cytometry follows our previous protocols (Park et al. 2020, 2021). Data acquisition was followed by analysis using the IDEAS software version 6.1.822.0 (Luminex Corporation). Analysis of bee hemolymph was performed on 10,000 events (cells) at a flow rate setting “low” and 60 × magnification. The gating strategy for honey bee hemocyte analysis is presented in Fig. 2.

## Results

During our experiment, the bees consumed the experimental feed with no significant difference from the control feed. The average consumption for the experimental group was 37.2 mL/7 days and 39.52 mL/7 days for the control group. The effect of HS on the selected immune parameters of *A*. *mellifera* was evaluated.

### Relative gene expression

**Fig. 3:**
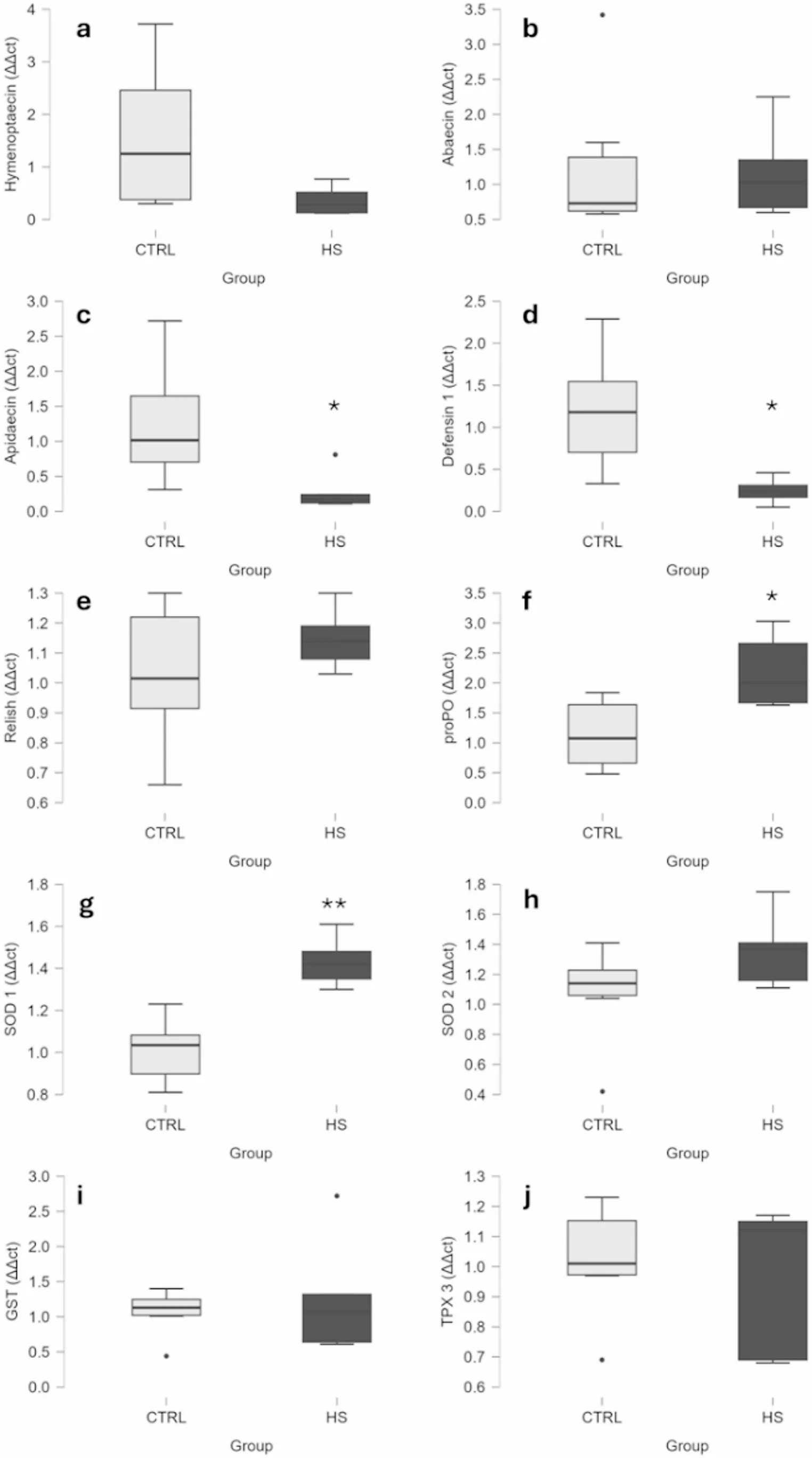
Relative expression level of (**a**) *hymenoptaecin*, (**b**) *abaecin*, (**c**) *apidaecin*, (**d**) *defensin 1*, (**e**) *relish*, (**f**) *proPO*, (**g**) *sod1*, (**h**) *sod2*, (**i**) *gst* and (**j**) *tpx3*, in the gut of bees receiving 0.5% of humic substances (*n* = 6), CTRL – control group, HS – Humic substances group. The number of stars indicates the level of significance (*p < 0.05, **p < 0.01). Statistical analysis was performed using the Student’s unpaired t- test

The relative expression of the genes *sod1*, *sod2*, *gst*, *tpx3*, *proPO*, *relish*, *abaecin*, *apidaecin*, *hymenoptaecin*, and *defensin 1* in the bee guts was evaluated to study the effect of HS on the immune response. A significant increase in the relative gene expression of *sod1* (*p* < 0.01, Fig. 3g) was observed in the HS group compared to the CTRL, and a significant increase was also observed in the expression of the *proPO* gene (*p* < 0.05, Fig. 3f). In contrast, a significant decrease was observed in the relative expression of the antimicrobial peptide genes *apidaecin* (*p* < 0.05, Fig. 3c) and *defensin 1* (*p* < 0.05, Fig. 3d) in the HS group. HS did not significantly influence the expression of *sod2* (Fig. 2h), *gst* (Fig. 3i), *tpx3* (Fig. 3j), *relish* (Fig. 3e), *abaecin* (Fig. 3b), or *hymenoptaecin* (Fig. 3a).

### Relative quantification of AMPs (ELISA)

**Fig. 4:**
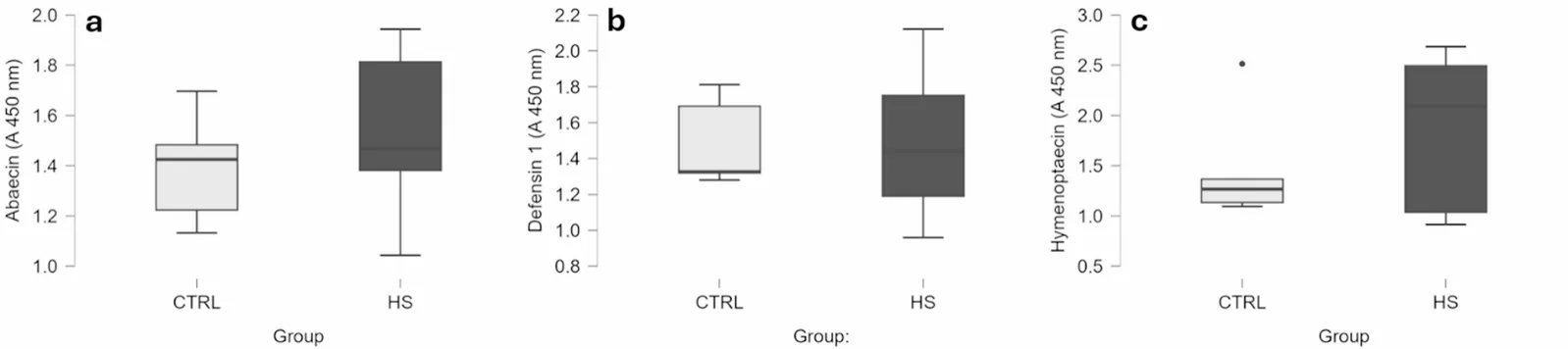
The effect of humic substances on relative quantification of (**a**) abaecin, (**b**) defensin 1, and (**c**) hymenoptaecin in hemolymph (*n* = 5). CTRL – control group, HS – Humic substances group. Statistical analysis was performed using the Student’s unpaired t- test

There were no significant changes after feeding with a 0.5% addition of HS on the levels of abaecin, hymenoptaecin and defensin 1 (Fig. 4a, b, c) in the hemolymph of bees.

### Quantification of lipid peroxidation

**Fig. 5:**
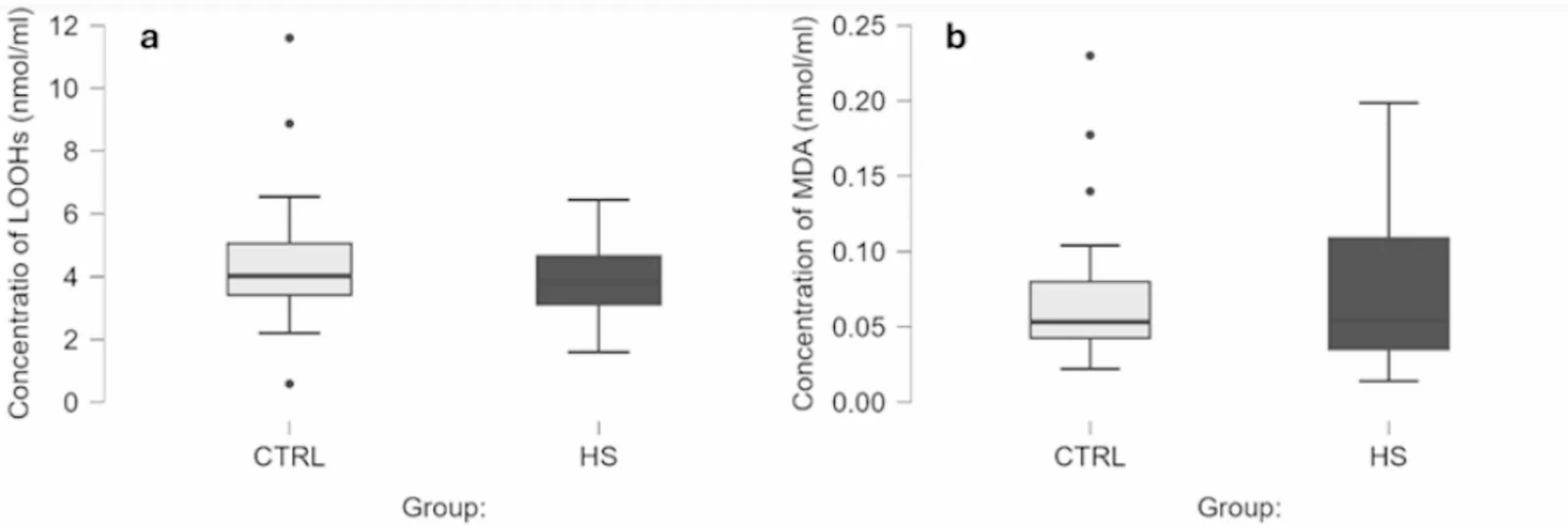
The effect of humic substances on lipid peroxidation. (**a**) The concentration of lipid hydroperoxides (LOOH) in [nmol⋅mL^− 1^], (**b**) Concentration of malondialdehyde (MDA) in [nmol⋅mL^− 1^], CTRL – control group, HS – Humic substances group (*n* = 30) in bee abdomen extracts. Statistical analysis was performed using the Mann–Whitney test (FOX2) and Student’s unpaired t-test (TBARS)

No significant changes in lipid peroxidation (Fig. 5a) and malondialdehyde (Fig. 5b) concentration were observed during the application of the 0.5% HS supplement.

### Determination of catalase activity and superoxide dismutase activity

**Fig. 6:**
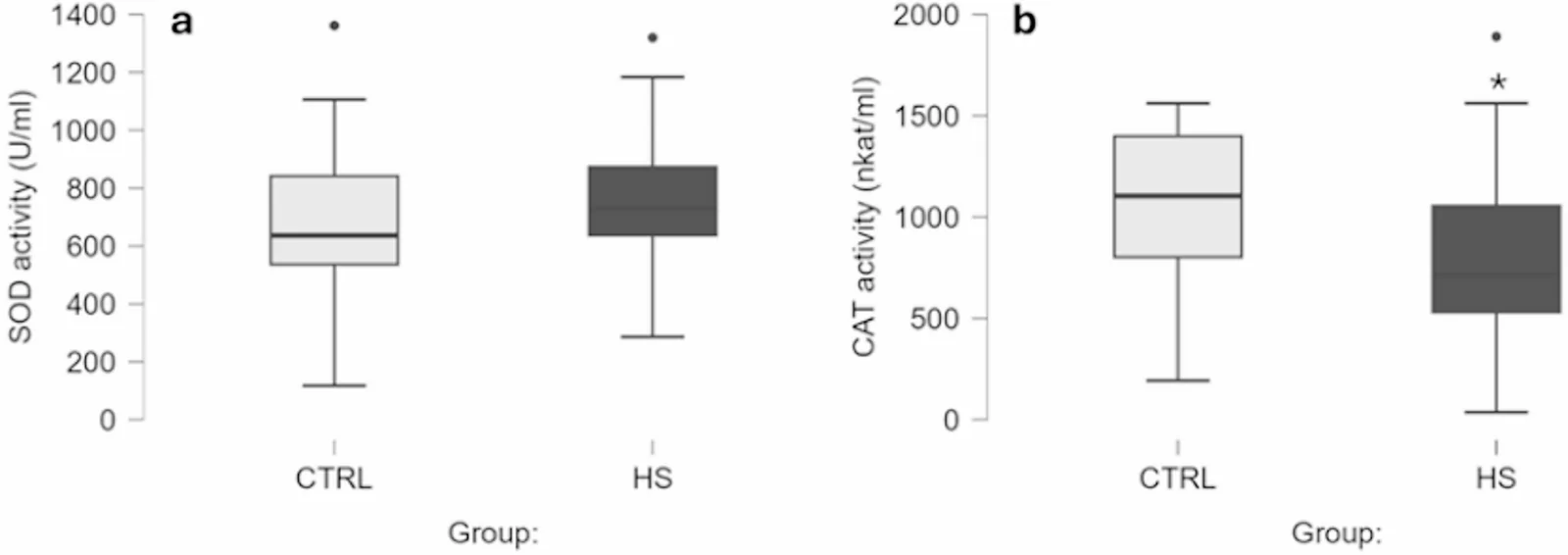
Enzyme activity of (**a**) SOD (U/mL) and (**b**) CAT (nkat/mL) in bee abdomen extracts. CTRL – control group, HS – Humic substances group, (*n* = 30). Statistical analysis was performed using the Mann–Whitney test. The number of stars indicates the level of significance (*p < 0.05)

As to the level of enzyme activity of the main enzymes of oxidative stress (SOD, CAT), we did not observe any significant differences in SOD activity in the HS-fed group compared to the control (Fig. 6a). On the other hand, we observed a significant decrease in CAT enzyme activity (Fig. 6b, *p* < 0.05) in the HS-fed group.

### Representation of hemocyte populations

**Fig. 7:**
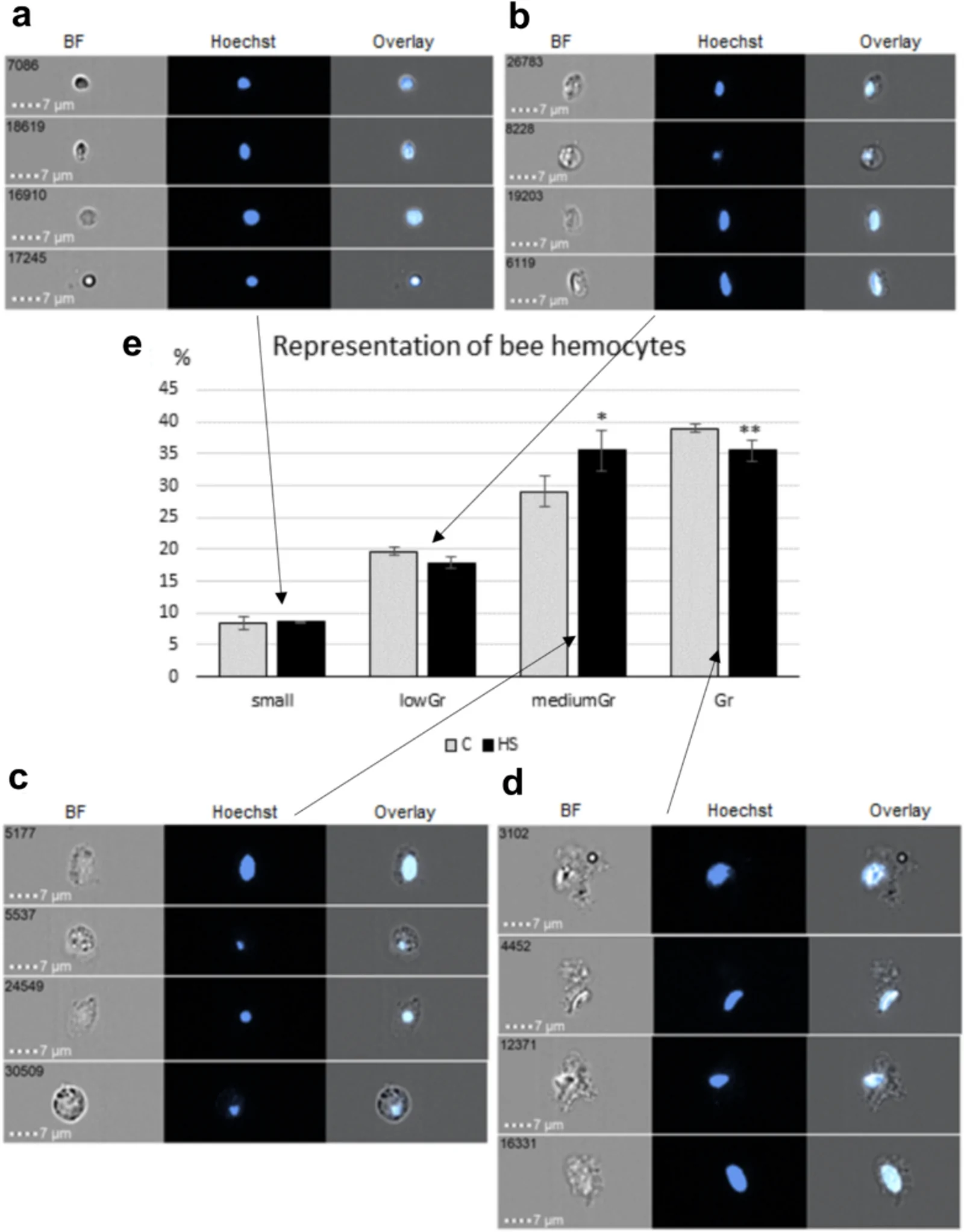
Influence of application of humic substances (HS) on the representation of bee hemocytes and the representative photos of: (**a**) small and very low granular cells, (**b**) low granular and medium cells, (**c**) medium granular and larger cells, and (**d**) high granular cells in hemolymph. The graph (**e**) shows differences in hemocyte representation between HS-treated and control honey bees. Significant differences between the HS group and the control group are marked with stars: * - *p* < 0.05; ** - *p* < 0.01

To study the effect of HS on hemocytes, imaging flow cytometry was used. The cells were divided based on their size and granularity into a population of small and very low granular cells, where prohemocytes are mainly represented (Fig. 7a). The “low GR” population contains cells with low granularity and a slightly larger size. Based on the photographs, this population consists mainly of plasmocytes and oenocytoids with low granularity (Fig. 7b). The “medium GR” cell population consists of medium granular and larger cells, either round in shape with mostly eccentrically located nuclei, granulocytes and probably granular oenocytoids or cells with oval nuclei morphologically similar to plasmocytes (Fig. 7c). The granular cell population contains cells of various sizes, but with high granularity, with the majority of the population being irregularly shaped cells with processes. A much smaller part of the population is made up of round cells with large round nuclei and a smaller amount of cytoplasm (Fig. 7d). By comparing the above hemocyte populations, we found that after HS application to bee colonies, there was a significant increase in the representation of medium granular cells (*p* < 0.01) at the expense of high granular cells (*p* < 0.05) and partially, but non- significantly, at the expense of low granular cells (Fig. 7e).

## Discussion

In this study, we examined the effect of a commercial HS product on selected immune genes, detoxification mechanisms and hemocyte populations in honey bees.

Antimicrobial peptides responsible for the humoral immunity of honey bees are produced mainly in the fat body during bacterial or fungal infection and also after exposure to xenobiotics (Pinďáková et al. 2025). Similarly, the response of AMPs (apidaecin, hymenoptaecin, abaecin, and defensin 1) to bacterial infection varies depending on whether the bee is exposed to Gram-positive or Gram-negative bacteria (Arafah et al. 2019). Decreased expression of AMP was observed during some viral infections (e.g. deformed wing virus) or exposure to neonicotinoids that suppress the immune system (Pluta and Sokół 2020). In our study, decreased gene expression of *apidaecin* and *defensin 1* was recorded in bees that received HS compared to the control, indicating a suppression of the immune response at the mRNA level. However, this decrease in gene expression did not translate into decreased protein levels, and therefore, the HS does not appear to functionally suppress humoral immunity under these conditions. However, it has to be noted that in the current work, gene expression was measured at the intestinal level to test local influence of perorally administered HS and protein levels were measured in hemolymph representing systemic immune answer, what could possibly cause the observed difference. The disconnect between gene and protein levels may also reflect post-transcriptional regulatory mechanisms or the stability of existing AMP proteins, which can persist in honey bee hemolymph for more than 8 days (Pinďáková et al. 2025).

In addition to antimicrobial peptides, we examined *prophenoloxidase* (*proPO*), a melanisation response marker. This gene encodes a key enzyme involved in melanin synthesis, which plays a crucial role in immune response and wound healing of bees (Zufelato et al. 2004). In our study, *proPO* gene expression was significantly increased in the HS-treated group compared to the control, suggesting that HS may enhance the immune response of bees. This contrasts with other substances used in bee nutrition, such as essential oils - thymol and carvacrol, which decreased the expression of the *proPO* in worker bees of the African *Apis mellifera* lineage (Canché-Collí et al. 2021). Additionally, environmental stressors like poor air quality and extreme temperatures reduce *proPO* expression as well (Mayack et al. 2023). Whether this observed transcriptional increase translates to enhanced enzyme activity requires further verification.

Antioxidant enzymes are critical for honey bee health, protecting them against reactive oxygen species (ROS) (Moreira et al. 2025). ROS are constantly produced in insect tissues during metabolic processes and mitochondrial respiration (Sies et al. 2017), but also in response to various stress factors such as pesticides, toxins, and pathogens (Tlak Gajger and Cvetkovikj 2025). In honey bees, superoxide dismutase and catalase are key components of the antioxidant defence system. In the present study, the relative gene expression of *sod1* was significantly increased in the intestine of honey bees, indicating the activation of the local antioxidant mechanisms upon the inclusion of HS in the diet. However, this increase did not translate to elevated SOD1 enzymatic activity measured in dissected abdomens without intestines. A similar gene–protein discordance, as observed for antimicrobial peptides, may be attributed, on one hand, to the use of different tissues for gene expression and protein analyses. On the other hand, changes detected at the local intestinal level in terms of gene expression may not have been reflected in the protein response assessed at the systemic level, namely in the hemolymph or in abdominal tissues devoid of the gut. The activity of CAT was decreased in the experimental group, which could result from adaptive downregulation or direct inhibition by HS. Expression of other detoxifying enzyme genes (*gst*, *tpx3*) also remained unchanged, suggesting that post-transcriptional or post-translational mechanisms may regulate enzymatic activity in response to HS.

To assess whether these enzyme changes affected cellular oxidative status, we measured lipid peroxidation markers. Despite the decreased CAT activity, overall antioxidant defence remained intact. This is evidenced by the measurement of MDA levels, which revealed no significant changes, nor did lipid hydroperoxide, assessed via the TBARS method. Lipid peroxidation is a commonly used biomarker of oxidative stress and cell membrane damage (Li-Byarlay et al. 2016; Simone-Finstrom et al. 2016). Toxic substances, including insecticides, herbicides, and heavy metals, can disrupt lipid peroxidation and antioxidant defence mechanisms in bees by inducing oxidative stress and impairing enzymatic antioxidant systems (Jumarie et al. 2017; Li et al. 2024; Mackei et al. 2025). Based on our results, HS do not disrupt oxidative balance within the bee organism. However, the continuous 7-day supplementation protocol used in this study may not represent the biologically optimal application strategy. Future studies should compare continuous versus pulsed supplementation regimens and test shorter application durations to identify protocols that maximise beneficial effects while minimising adaptive responses.

Variation in hemocyte types and the lack of specific cellular markers have constrained efforts to develop a universally applicable insect hemocyte classification system. To date, there is no universally accepted classification of hemocytes even within the Apidae family or the genus *Apis*. The existing classification schemes differ substantially depending on the criteria used, including morphological features, cellular functions, or the use of specific molecular or immunological markers (Marringa et al. 2014; Richardson et al. 2018; Gábor et al. 2020). For hemocyte identification, we employed imaging flow cytometry, which enables rapid analysis of individual cells from small sample volumes using fluorescent dyes. This approach allows cells to be classified not only based on size, granularity, and fluorescence intensity, but also enables detailed morphological characterisation from acquired images, thereby improving and refining the gating strategy. Based on these characteristics, the hemocytes were categorised into 4 populations. The smallest and least granular cells primarily correspond to prohemocytes. Slightly larger cells with low granularity, which, according to imaging, mainly consist of plasmocytes and low granularity oenocytoids. Cells with intermediate granularity and larger size included round cells with eccentrically located nuclei, granulocytes and likely granular oenocytoids, as well as cells with oval nuclei resembling plasmocytes. Lastly, highly granular cells of varying size, with most exhibiting irregular shapes and cytoplasmic extensions – typical granulocytes, while a smaller fraction consisted of round cells with large nuclei and relatively little cytoplasm. Findings of Marringa et al. (2014) demonstrated a surprisingly high variability in hemocyte profiles among honey bees originating from the same hive. Such heterogeneity may be partly explained by age-dependent shifts in hemocyte composition, which naturally occur during adult insect development. Additionally, temporal changes in hemocyte dynamics may reflect variation in colony conditions or responses to external stressors, including environmental factors, chemical exposure, or pathogen challenge.

Based on the above-defined classification of hemocyte populations, we compared the effect of HS on their relative representation. Following the HS application, we observed a significant increase in cells with intermediate granularity at the expense of highly granular cells. Given that the stimulatory effects of HS on phagocytic activity have been repeatedly demonstrated in other animal species (Islam et al. 2005; Mudroňová et al. 2020, 2021; Zykova et al. 2025), it can be assumed that HS may exert a similar effect in bees. Granulocytes and plasmatocytes, which exhibit phagocytic activity in bees, are included within the population of cells of intermediate granularity (Hystad et al. 2017). This population also comprises oenocytoids, which produce phenoloxidase, an enzyme involved in melanin formation during the melanisation process (Ashida et al. 1988). Consistently, we recorded a significant increase in prophenoloxidase gene expression in the HS-treated group, supporting this association. To conclusively confirm the effect of HS on cellular immunity in bees, particularly on phagocytosis, future experiments should assess not only the relative abundance of hemocytes but also their functional activity.

## Conclusions

This study demonstrated that 7-day continuous supplementation with 0.5% humic substances modulated honey bee immune parameters without adverse effects under laboratory conditions. HS increased *sod1* and *proPO* gene expression while decreasing *apidaecin* and *defensin 1* expression on the local gut level, although these transcriptional changes did not consistently translate to altered protein or enzyme activity levels evaluated as a systemic answer in the hemolymph or the bee abdomens. CAT activity decreased significantly, yet oxidative damage markers remained unchanged, indicating maintained redox homeostasis. HS also altered hemocyte composition. While HS supplementation at 0.5% appears safe for healthy bees, future research should include time-course studies, dose-response experiments, comparison of supplementation regimens, pathogen challenge trials, and field validation to determine whether HS can serve as an effective health-promoting additive in apiculture.

## Data Availability

The original contributions presented in this study are included in the article. Further inquiries can be directed to the corresponding authors.
